# Treatment of Cancer of the Rectum

**Published:** 1885-01

**Authors:** 


### Treatment of Cancer of the Rectum.

In a clinical lecture at the Necker Hospital, Professor Trélat drew the following conclusions with regard to the treatment of cancer in the rectum:

1. Cancers of the rectum should not be touched, unless they cause grave disorders. This rule should be positive, with the single exception that very small cancerous deposits may be removed from the lower part of the rectum and the margin of the anus.

2. In all other cases the treatment should be confined to complications and palliative operations. In giving these rules I am in accord with Professor Verneuil.

3. As palliative operations, rectotomy may be done when the finger can be passed beyond the upper limit of the neoplasm. If the neoplasm is more extensive, the surgeon should abandon rectotomy, and work out a way of derivation; for by performing rectotomy in these cases, the surgeon is almost certain to injure the peritoneum. With the English surgeons, and Labbê and Tillaux, I am in favor of lumbar colotomy, because it is a simple operation, less dangerous, and affords a ready means of exit for the feces. Other surgeons prefer to make an inguinal anus; but there is risk of opening the small intestine, with all the attendant dangers and inconveniences.—*Revue de Thérap.*

